# Left atrial appendage closure: Current status, unresolved issues, and future perspectives

**DOI:** 10.1007/s12928-025-01222-6

**Published:** 2025-12-11

**Authors:** Yusuke Kondo, Satoko Ryuzaki, Miyo Nakano, Yoshio Kobayashi

**Affiliations:** 1 Department of Cardiovascular Medicine, Chiba University Graduate School of Medicine, Chiba, 260-8670 1-8-1 Inohana, Chuo-ku Japan; 2 Department of Advanced Arrhythmia Bioengineering, Chiba University Graduate School of Medicine, Chiba, Japan; 3 Department of Advanced Cardiorhythm Therapeutics, Chiba University Graduate School of Medicine, Chiba, Japan

**Keywords:** Left atrial appendage closure (LAAC), Atrial fibrillation (AF), Non-valvular atrial fibrillation (NVAF), Stroke

## Abstract

Left atrial appendage closure (LAAC) is an established therapy for stroke prevention in patients with nonvalvular atrial fibrillation (AF) who cannot tolerate long-term oral anticoagulation (OAC). In Japan, LAAC was introduced in 2019 and has been performed in more than 10,000 cases a0s of 2025. Although indications remain limited to high-risk patients with OAC contraindications, procedural volume continues to rise. This review outlines the current status, remaining challenges, and future perspectives of LAAC, with emphasis on the WATCHMAN device. The WATCHMAN FLX and FLX Pro have enhanced procedural safety, sealing efficacy, and anatomical adaptability. Large clinical trials and registries have confirmed stroke prevention efficacy comparable to OAC with fewer hemorrhagic complications. However, device-related thrombus (DRT) and peri-device leak (PDL) remain major concerns, underscoring the need to optimize postprocedural antithrombotic therapy. The Amulet device provides superior ostial sealing through its dual-disk design but is technically more complex and associated with higher procedural complication rates. Intracardiac echocardiography (ICE) has emerged as a less invasive alternative to transesophageal echocardiography, reducing anesthesia requirements and enabling same-session LAAC with AF ablation. The development of pulsed field ablation (PFA) further improves the feasibility of such integrated approaches. Future priorities include risk stratification for DRT, individualized antithrombotic strategies, and broader indications encompassing OAC-resistant embolic stroke and other high-risk populations. With advancing technology, ICE-guided, minimally invasive workflows and integrated ablation–occlusion procedures are expected to establish LAAC as a key component of comprehensive AF management.

**Graphical Abstract:**

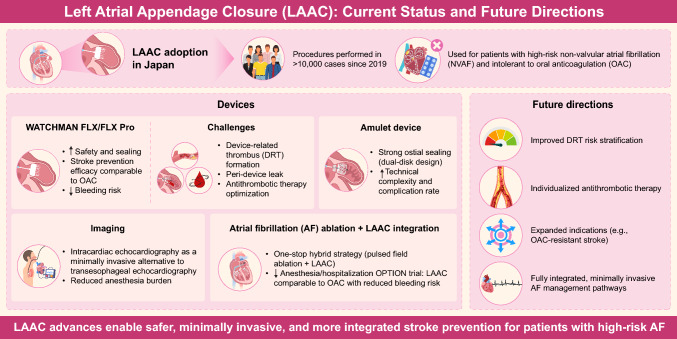

## Background and rationale

Atrial fibrillation (AF) is the most common sustained cardiac arrhythmia, and its prevalence increases markedly with age. In Japan, AF affects approximately 10% of individuals aged ≥ 70 years and nearly 15% of those aged ≥ 80 years [1–3]. With the demographic shift toward an aging society, the number of patients with AF is expected to rise further [4, 5]. Although often asymptomatic, AF has major clinical implications as it contributes to worsening heart failure, cognitive decline, and—most critically—cardioembolic stroke [6–20].

In Japan, catheter ablation procedures to reduce AF burden have increased annually, making ablation a major therapeutic option [21–23]. In nonvalvular AF, the left atrial appendage (LAA) is the main site of thrombus formation. Percutaneous LAA closure (LAAC) has become an established treatment for patients in whom long-term oral anticoagulation (OAC) is contraindicated or not tolerated [6, 7]. The SALUTE trial (Study of Left Atrial Appendage Closure for Nonvalvular Atrial Fibrillation Using the WATCHMAN Device in Japan) led to the regulatory and insurance approval of the WATCHMAN device in 2019 [24, 25]. By August 2025, more than 10,000 procedures had been performed nationwide. Representative Japanese studies, including two major registries—the TERMINATOR and OCEAN-LAAC registries—are summarized in Table 1 [24–31, 33–48].

**Table 1:** Representative clinical studies on LAAC conducted in Japan to date

No	Publication year	Study period / No. of centers	Sample size	Device	Study design	Specific population	Key outcomes	Reference
1	2018	2017 / 10 centers	54 (ITT cohort 42)	WATCHMAN 2.5	Prospective single-arm (SALUTE trial)	NVAF patients unsuitable for long-term OAC	Implant success 100%; one ischemic stroke (2.4%) during 6-month follow-up	Aonuma K, et al. Circ J 2018;82:2946–2953 [24]
2	2020	2017 – 2019 / 10 centers	55 (ITT cohort 42)	WATCHMAN 2.5	Follow-up cohort of SALUTE	NVAF patients enrolled in SALUTE	Effective LAAC 100%; 3 ischemic stroke (7.1%) and one death (2.4%) during 24-month follow-up	Aonuma K, et al. Circ J 2020;84:1237–1243 [25]
3	2022	2020 – 2021 / single center	93	WATCHMAN FLX vs 2.5	Retrospective observational study	NVAF patients (real-world)	Implant success FLX 100% vs 2.5 98%; no PDL > 3 mm with FLX; similar short-term safety	Fukuda N, et al. J Clin Med 2022;11:1618 [26]
4	2022	2019 – 2020 / single center	55	WATCHMAN 2.5	Retrospective observational study	NVAF patients (initial implant era)	Implant success 98.2%; no procedural complications; DRT 7.1% (1 stroke-related); major bleeding in 21%	Fukunaga M, et al. J Cardiol 2022;79:752–758 [27]
5	2023	2019 – 2021 / 19 centers	548	WATCHMAN (2.5/FLX)	Prospective study (OCEAN-LAAC registry)	NVAF patients (mean age 76 y)	Implant success 96.5%; OAC discontinuation 90% at 45 days; higher early bleeding in elderly	Asami M, et al. JACC Asia 2023;3:272–284 [28]
6	2023	2020 – 2022 / single center	118	WATCHMAN (2.5/FLX)	Retrospective observational study	NVAF patients including HD patients	Implant success 100%; major complications rare; feasible even in HD patients	Ueno H, et al. Cardiovasc Interv Ther 2023;38:410–420 [29]
7	2023	2020 – 2022 / single center	74	WATCHMAN (2.5/FLX)	Retrospective observational study	NVAF patients (real-world)	Implant success 98.6%; DRT 13% in one group; major bleeding 6% vs 2% vs 9%	Ryuzaki S, et al. Circ J 2023;87:1820–1827 [30]
8	2023	2021 – 2023 / single center	15	WATCHMAN FLX	Retrospective observational study	CKD stage 3b–5 patients undergoing contrast-free LAAC (excluding HD)	Contrast-free LAAC safe and feasible; no periprocedural complications; no acute kidney injury at 45 days	Fukushima T, et al. Circ J. 2023;88:170–174 [31]
9	2023	2019 – 2023 / single center	45	WATCHMAN (2.5 / FLX)	Retrospective observational study	NVAF patients undergoing LAAC, 42% with prior gastrointestinal bleeding (GIB)	Prior GIB predicted higher post-LAAC bleeding; 7/8 post-LAAC bleeds were GIB	Kikuchi T, et al. JGH Open. 2023;8:e13009 [33]
10	2024	2021 – 2023 / single center	141	WATCHMAN FLX	Retrospective observational study	CKD patients undergoing contrast-free LAAC	Contrast-free LAAC safe and feasible; no DRT/PDL > 3 mm; stable eGFR	Okada A, et al. Cardiovasc Interv Ther. 2024; 39: 191–199 [34]
11	2024	2019 – 2022 / 19 centers	729	WATCHMAN	Prospective multicenter registry (TERMINATOR registry)	NVAF patients (real-world)	Implant success 99%; stroke 2.2%; major bleeding 3.7%; composite CV death/stroke/SE 3.4%	Hara H, et al. J Cardiol. 2024;83:298–305 [35]
12	2024	– / single center	46	WATCHMAN (2.5 / FLX)	Retrospective observational study	NVAF patients undergoing both LAAC and catheter ablation within two years	No new DRT or PDL after CA in LAAC-first group; 3 new PDLs after CA-first approach	Chatani R, et al. J Arrhythmia 2024;40:879–890 [36]
13	2024	2019 – 2022 / 19 centers	1,464	WATCHMAN FLX vs 2.5	Prospective registry study (OCEAN-LAAC registry)	NVAF patients (real world)	Implant success FLX 95.8% vs 2.5 91.9%; 1-year bleeding 7.8% vs 16.4%	Nakashima M, et al. Circ J 2024;88:1187–1197 [37]
14	2024	2019 – 2023 / 20 centers	225	WATCHMAN (2.5 / FLX)	Prospective study (OCEAN-LAAC registry)	NVAF patients with LAVI data at baseline and 6 months	LAVI increase linked to smaller baseline LAVI and higher TRPG; LAVI rise predicted HF hospitalization	Nonaka H, et al. Int J Cardiol Heart Vasc. 2024;53:101,449 [38]
15	2024	2019 – 2021 / 4 centers	63	WATCHMAN	Retrospective observational study	NVAF patients evaluated by CT-based virtual TEE (V-TEE) vs TEE	V-TEE larger; correlates well; better for elliptical LAA	Cho N, et al. Heart Vessels. 2024; 39: 539–548 [39]
16	2024	2019 – 2022 / 2 centers	199	WATCHMAN FLX vs 2.5	Retrospective observational study	NVAF patients (real-world)	Shorter procedure time (42 vs 50 min); tamponade 0 vs 5.6%; complete seal 80% vs 63%; 1-year DRT 7.0% vs 3.4%	Chatani R, et al. Catheter Cardiovasc Interv 2024;104: 318–329 [40]
17	2024	2019 – 2022 / 19 centers	937	WATCHMAN (2.5 / FLX)	Prospective study (OCEAN-LAAC registry)	NVAF patients with available baseline BNP levels (excluding HD)	Higher baseline BNP independently predicted stroke/bleeding or death after LAAC	Imamura T, et al. J Clin Med. 2024;13:6232 [41]
18	2024	2019 – 2022 / single center	143	WATCHMAN (2.5 / FLX)	Retrospective observational study	NVAF patients categorized by prior thromboembolism	Preprocedural stroke predicted post-LAAC DRT/TE; extended OAC lowered risk without added bleeding	Sumiyoshi H, et al. Heart Vessels. 2024;39:1045–1059 [42]
19	2025	2019 – 2022 / 19 centers	1,464	WATCHMAN (2.5 / FLX)	Prospective study (OCEAN-LAAC registry)	NVAF patients undergoing LAAC stratified by DOAC score (high vs low bleeding risk)	HBR group had higher 1-year events (17.6% vs 12.4%), mainly bleeding; DOAC score outperformed HAS-BLED	Asami M, et al. CJC Open. 2025; 7: 420–428 [43]
20	2025	2019 – 2022 / 19 centers	1,464	WATCHMAN (2.5 / FLX)	Prospective study (OCEAN-LAAC registry)	NVAF patients with and without HD	LAAC feasible in HD; similar procedural success (97%) and stroke rate vs non-HD	Tanaka S, et al. JACC Asia. 2025; 5: 174–186 [44]
21	2025	2019 – 2022 / 19 centers	1,350	WATCHMAN (2.5 / FLX)	Prospective study (OCEAN-LAAC registry)	NVAF patients undergoing LAAC with or without angiographic residual trabeculation	Residual trabeculation in 5.6%; associated with higher PDL at procedure, but no difference in DRT or stroke at follow-up	Chatani R, et al. J Cardiovasc Electrophysiol. 2025;36:347–358 [45]
22	2025	2019 – 2022 / single center	238	WATCHMAN (2.5 / FLX)	Retrospective observational study	NVAF patients with malignant LAA (stroke or thrombus despite OAC)	LAAC alone had higher stroke risk; LAAC + continued OAC may reduce events	Chatani R, et al. Heart Rhythm 2025; 22: 475–485 [46]
23	2025	2020 – 2023 / single center	192	WATCHMAN (2.5 / FLX) + MitraClip	Retrospective observational study	NVAF patients undergoing LAAC, with or without concomitant TEER for MR	Combined LAAC + TEER feasible; similar procedural success and 1-year outcomes vs LAAC alone	Fukuda N, et al. Cardiovasc Interv Ther. 2025;40: 400–413 [47]
24	2025	2019 – 2022 / single center	1,397	WATCHMAN (2.5 / FLX)	Prospective study (OCEAN-LAAC registry)	Presence/absence and degree of peridevice leak (PDL) at implantation	PDL present in 6.0%; PDL associated with higher risk of TIA/IS/systemic embolism (adjusted sHR 4.25 [95% CI 1.91–9.44], *P* < 0.001); even PDL ≤ 3 mm increase	Saito T, et al. J Am Heart Assoc. 2025;14: e044422 [48]

*AF* atrial fibrillation, *BNP* B-type natriuretic peptide, *CA* catheter ablation, *CKD* chronic kidney disease, *CV* cardiovascular, *DOAC* direct oral anticoagulant, *DRT* device-related thrombus, *eGFR* estimated glomerular filtration rate, *GIB* gastrointestinal bleeding, *HBR* high bleeding risk, *HD* hemodialysis, *ITT* intention-to-treat, *LAAC* left atrial appendage closure, *LAA* left atrial appendage, *LAVI* left atrial volume index, *MR* mitral regurgitation, *NVAF* nonvalvular atrial fibrillation, *OAC* oral anticoagulant, *PDL* peri-device leak, *sHR* sub-hazard ratio, *TEER* transcatheter edge-to-edge repair, *TIA* transient ischemic attack, *TRPG* tricuspid regurgitation pressure gradient, *V-TEE* virtual transesophageal echocardiography

However, the indication for LAAC in Japan remains limited. According to domestic consensus, LAAC is considered for high-risk patients with nonvalvular AF who have an elevated risk of stroke or systemic embolism based on the CHADS₂ or CHA₂DS₂-VASc score. It is recommended for those who cannot safely continue OAC due to one or more of the following: HAS-BLED score ≥ 3, recurrent fall-related trauma, cerebral amyloid angiopathy, prolonged (≥ 1-year) dual antiplatelet therapy, or major bleeding (BARC type 3, Class IIb) [7]. Thus, LAAC is reserved for patients “for whom OAC is indicated but cannot be safely maintained.”

In contrast, in the United States, LAAC for patients unable to maintain long-term OAC is a Class IIa recommendation, giving institutions greater discretion in patient selection [49]. Although such restrictions have slowed adoption in Japan, the number of procedures continues to increase steadily, reflecting growing clinical acceptance [26–48].

Recent advances include diversification of available devices (with the introduction of Amulet in addition to WATCHMAN), expansion of imaging guidance from transesophageal echocardiography (TEE) to intracardiac echocardiography (ICE), and growing use of combined procedures with AF ablation. This review summarizes the current status and challenges of the WATCHMAN device, outlines the characteristics and safety considerations of Amulet, examines the evolving role of ICE guidance, and discusses emerging strategies such as combined LAAC and ablation, unresolved issues, and future perspectives.

## Current status and challenges of the WATCHMAN device

The WATCHMAN device is the most representative system establishing percutaneous LAAC as a clinically validated therapeutic strategy. Since the first-generation WATCHMAN, continual refinements have led to widespread adoption of the WATCHMAN FLX and FLX Pro. The device features a self-expanding nitinol frame with distal anchors securing it within the LAA for stable fixation. The proximal surface is covered with a polyethylene terephthalate (PET) fabric that seals the cavity and prevents thrombus formation. Recent designs employ a fully closed distal end to enhance recapturability, procedural safety, and anatomical compatibility.

The efficacy and safety of LAAC have been demonstrated through randomized trials, large registries, and post-marketing studies over the past two decades. In Japan, the first approved device was the WATCHMAN 2.5, followed by the WATCHMAN FLX and FLX Pro. The pivotal PROTECT AF trial showed that the WATCHMAN 2.5 was noninferior to warfarin for preventing thromboembolic events, and the PREVAIL trial confirmed these findings [50–52]. Together, they established that the WATCHMAN device provides stroke prevention efficacy comparable to warfarin while reducing hemorrhagic complications.

In 2020, the PRAGUE-17 trial compared LAAC (using WATCHMAN 2.5, WATCHMAN FLX, or Amulet) with direct oral anticoagulant (DOAC) therapy and demonstrated noninferiority for the composite endpoint of stroke, systemic embolism, or major bleeding [53]. This evidence confirmed that LAAC is an effective alternative to both warfarin and modern DOAC therapy. The U.S. Food and Drug Administration (FDA) approved the WATCHMAN 2.5 in 2015, and subsequent post-approval and real-world studies verified its long-term efficacy and safety [49].

Five-year combined results from PROTECT AF and PREVAIL showed that LAAC achieved outcomes equivalent to warfarin for the composite endpoint of stroke, systemic embolism, and cardiovascular or unexplained death, while significantly lowering rates of hemorrhagic stroke and major bleeding [52]. Over time, this translated into lower cardiovascular and all-cause mortality, indicating cumulative benefit with prolonged follow-up.

The next-generation WATCHMAN FLX was designed to further improve procedural safety and efficacy, playing a central role in global LAAC expansion. Compared with earlier versions, the FLX offers a wider size range, shallower depth, polymer coating to minimize metal exposure, an 18-strut dual-row anchor frame, and a closed-end design for improved stability and sealing. The “ball-shaped” configuration reduces sharp edges, allowing safer manipulation within the LAA. In the PINNACLE FLX trial, complete LAA closure was achieved in 100% of patients, peridevice leaks (PDLs) were ≤ 5 mm at one year, and device-related thrombus (DRT) rates were markedly lower [54]. Real-world data from the NCDR SURPASS registry, comprising more than 16,000 cases, reported a 97.6% procedural success rate and 0.37% major complication rate, confirming excellent safety and performance [55].

Following FDA approval in July 2020, WATCHMAN FLX adoption grew rapidly, surpassing 300,000 implantations by 2023. The WATCHMAN FLX Pro, introduced in September 2023, features a polymer coating to promote endothelialization, enhanced fluoroscopic visibility, and a larger 40 mm size for broader anatomical suitability. Due to the strong performance of the FLX, the FLX Pro has yet to be widely introduced in several European countries. These technological improvements have enabled LAAC to achieve outcomes comparable or superior to OAC across short-, mid-, and long-term follow-up.

Despite these advances, several challenges persist. The most important is DRT, which usually develops within the first few months after implantation, with an incidence of approximately 3–4% [56, 57]. DRT is a strong predictor of thromboembolic events, making prevention and early detection essential for long-term safety. Reported risk factors include enlarged left atrium, reduced left atrial ejection fraction, incomplete closure, diabetes, and advanced age. Optimization of post-procedural antithrombotic therapy and imaging follow-up is therefore critical.

PDL is another concern. Because the WATCHMAN anchors deeply within the LAA, it may not fully conform to complex ostial morphologies, leaving small residual leaks. The clinical relevance of minor PDLs remains debated—some seal spontaneously through endothelialization, whereas larger leaks may increase thromboembolic risk and require re-intervention.

Finally, the optimal post-procedural antithrombotic regimen remains under discussion. The FDA-approved protocol initially combined warfarin with aspirin for 45 days, followed by dual antiplatelet therapy (DAPT) and then single antiplatelet therapy (SAPT). However, clinical practice has shifted toward DOAC-based regimens and shorter DAPT durations, with newer strategies such as SAPT alone or low-dose DOAC combinations for patients at high bleeding risk [58, 59]. These variations significantly influence patient selection, outcomes, and international comparability, highlighting the need for global standardization and region-specific optimization of antithrombotic management.

## Characteristics and challenges of the amulet device

The Amplatzer Amulet (Abbott) represents the second major LAAC device following the WATCHMAN, and it became commercially available in Japan in 2025. Derived from the earlier Amplatzer platform designed for atrial septal defect (ASD) or patent foramen ovale (PFO) closure, the Amulet employs a dual-seal design composed of a distal plug and proximal disc [60–63]. The plug anchors within the LAA, while the disc covers the ostium “surface-wise,” enhancing sealing and mechanical stability. This configuration enables effective closure even in shallow or morphologically complex LAAs [62, 63].

The Amulet offers a broad size range (16–34 mm) and a shorter length, accommodating wide anatomic variation. The plug has both proximal and distal anchors, and the disc provides complete ostial coverage, resulting in a more “surface-occluding” mechanism compared with the deep-anchoring design of the WATCHMAN. The Amulet often achieves better sealing in chicken-wing or multilobed appendage anatomies; however, its structure can make disc–plug axis alignment technically demanding. Because its recapture flexibility is lower than that of the WATCHMAN, precise initial positioning is essential. Inexperienced operators tend to have longer procedures and higher recapture rates.

Although the Amulet demonstrates excellent sealing performance, randomized trials and registries have shown a slightly higher incidence of procedural complications—particularly pericardial effusion and periprocedural bleeding—than with the WATCHMAN [64, 65]. In the Amulet IDE trial, efficacy was noninferior to the WATCHMAN, but early procedure-related complications occurred more often (2.9% vs. 1.6%), primarily due to pericardial effusion [66]. The SWISS-APERO trial also confirmed superior sealing with the Amulet but noted greater procedural complexity and more frequent recapture events compared with the WATCHMAN FLX [56]. Therefore, operator experience, detailed CT-based planning, and precise transseptal puncture are critical for procedural safety. When implanting the Amulet device, careful attention must be paid to the positional relationship between the pulmonary artery and the anchors.

Imaging assessment remains challenging. DRT occurs at similar frequency as with the WATCHMAN (approximately 3–4%); however, the thrombus location is more variable—often forming on the disc surface or plug lobes rather than the central hub [67]. Because thrombus on the disc may carry higher embolic potential, careful surveillance using TEE or cardiac CT is warranted. Metallic components of the Amulet can cause imaging artifacts that obscure small PDLs or thrombi, highlighting the need for standardized multimodality follow-up protocols.

Optimization of post-implant antithrombotic therapy also remains under investigation [68]. The traditional regimen—short-term DAPT followed by SAPT—is being replaced by individualized strategies. In high-bleeding-risk patients, shortened DAPT or SAPT alone, and in some cases low-dose DOAC combinations, are being explored [69]. These evolving regimens may reduce bleeding risk without compromising endothelialization, though additional randomized evidence is needed.

In summary, the Amulet provides excellent ostial sealing and wide anatomic applicability but presents challenges related to technical complexity, limited recapture flexibility, and imaging assessment. Continued refinement of procedural techniques, imaging modalities, and postoperative management will be essential to fully realize its clinical potential.

## Evolution of intra-procedural imaging modalities: From TEE to ICE

Intra-procedural imaging is crucial for the safety and efficacy of LAAC. Traditionally, TEE has been the standard imaging method. TEE provides high spatial resolution and Doppler capability, enabling detailed visualization of the LAA, device–wall relationships, and PDLs [69]. However, TEE requires general anesthesia or deep sedation, which may be burdensome for older adults or those with respiratory disease, esophageal pathology, or frailty. These requirements prolong recovery and increase resource utilization, prompting interest in less invasive imaging techniques [70].

ICE has emerged as a promising alternative. ICE catheters are introduced via femoral venous access and advanced into the right atrium or interatrial septum, allowing direct visualization of the LAA without airway manipulation [71]. This approach permits procedures under local anesthesia and conscious sedation, reducing anesthesia-related risks and recovery time. ICE has gained rapid acceptance, especially in centers experienced in structural heart interventions such as transcatheter ASD closure or atrial fibrillation ablation.

The ICE-LAA study, a prospective single-arm trial in 100 patients, demonstrated the feasibility of LAAC under 2D ICE guidance [72]. Complete closure was achieved in 98.5% of patients, with only 1.5% showing minor (3–5 mm) intra-procedural leaks. These findings underscored the ability of ICE to provide real-time visualization of LAA anatomy and device deployment. Multicenter experiences, including the ICE-TEE trial, confirmed similar procedural success and complication rates compared with TEE-guided LAAC, supporting broader clinical adoption [73].

Despite these advantages, ICE has inherent limitations. Its imaging field is narrower than that of TEE, making distal LAA lobes and micro-PDLs difficult to visualize [70, 71]. Moreover, manipulation of the catheter within the left atrium requires a learning curve, particularly for consistent imaging of device compression and positioning. Operator experience is therefore essential to avoid incomplete assessment.

Recent advances in three-dimensional (3D) and four-dimensional (4D) ICE technology have improved spatial orientation and depth perception, allowing multiplanar reconstruction comparable to TEE [71, 74, 75]. These next-generation systems, now under evaluation in Europe and the United States, may enable fully ICE-guided LAAC procedures without adjunctive TEE. Integration of ICE with fusion imaging systems using CT or fluoroscopy is also being explored to further improve accuracy and safety.

In Japan, national reimbursement approval for ICE-guided LAAC followed successful domestic feasibility trials in 2025. As ICE technology evolves, it is expected to become the standard imaging modality for minimally invasive LAAC, supporting same-day discharge protocols and combined procedures such as concomitant AF ablation and LAA closure.

## Combined AF ablation and LAAC

The combined procedure of AF ablation and LAAC has attracted growing interest due to its clinical practicality and procedural efficiency. Performing catheter ablation—aimed at eliminating AF—together with LAAC—designed to prevent thromboembolic events—within a single transseptal puncture and anesthesia session provides substantial advantages for patients. This approach is particularly beneficial for older adults and those with comorbidities, as it reduces repeated anesthesia exposure, hospitalizations, and cumulative procedural risk, while offering potential cost benefits [76].

In current clinical practice, the “ablation-first” strategy is most commonly adopted, wherein catheter ablation is completed before LAAC. This sequence stabilizes left atrial hemodynamics and minimizes thrombotic risk during device implantation. However, ablation induces transient edema and inflammation of the atrial wall, which can alter the LAA ostium morphology [77]. Therefore, intra-procedural reassessment of the LAA using TEE or ICE, in addition to pre-procedural CT planning, is essential for accurate device selection and positioning.

Anticoagulation management is another major consideration. After AF ablation, transient thrombotic risk necessitates continued OAC [78]. During LAAC, anticoagulation must be balanced to prevent bleeding complications such as pericardial effusion or vascular injury. Accordingly, timing and peri-procedural anticoagulation strategies should be individualized according to patient risk.

The pivotal OPTION trial compared LAAC after AF ablation with continued OAC therapy [78, 79]. LAAC proved noninferior for preventing stroke, systemic embolism, or cardiovascular death and resulted in significantly fewer major bleeding events. These findings support post-ablation LAAC as a safe and effective option for discontinuing long-term OAC in appropriately selected patients. It should be noted that the ablation techniques used in the OPTION trial were almost exclusively radiofrequency or cryoballoon ablation, and pulsed field ablation (PFA) was not included.

The introduction of PFA, a myocardial-selective ablation technology based on electroporation, has accelerated integrated treatment strategies for patients with AF [80–83]. Compared with conventional radiofrequency (RF) or cryoballoon ablation, PFA offers shorter procedural times, less inflammation-related edema, and a lower risk of collateral tissue injury [84, 85]. Its clinical adoption has expanded rapidly, supported by evidence demonstrating safety and efficacy [86, 87]. Importantly, PFA may enable shorter post-procedural anticoagulation, making it well suited for integration with LAAC [88]. Prospective studies in Europe, the United States and Asia are evaluating this “one-stop hybrid strategy” combining PFA and LAAC, with encouraging results showing reduced procedural time and complication rates.

Nevertheless, debate continues regarding whether ablation and LAAC should be performed simultaneously or in staged procedures [36, 89, 90]. In the near future, combined AF ablation and LAAC is expected to become an integrated therapeutic approach for patients unsuitable for long-term anticoagulation. This “one-puncture, one-anesthesia” strategy offers simultaneous rhythm control and embolic protection through a single, comprehensive intervention.

## Unresolved issues

Despite the increasing use of LAAC, several clinical and technical challenges persist. The foremost concern is DRT, which occurs in approximately 3–4% of cases and remains a major cause of stroke and systemic embolism [29, 56, 91, 92]. Multiple factors contribute to DRT formation, including left atrial enlargement, reduced atrial contractility, diabetes, advanced age, and suboptimal device positioning. Developing risk models that integrate atrial function with inflammatory and coagulation biomarkers represents a key next step for prevention and early detection.

Optimization of post-procedural antithrombotic therapy is another crucial issue. The conventional regimen—warfarin plus DAPT followed by SAPT—may not suit high-bleeding-risk patients. Contemporary strategies increasingly include shortened DAPT, SAPT alone, low-dose DOAC + SAPT, or low-dose DOAC regimens [6, 27, 28, 30, 35, 92]. Given the higher bleeding tendency in Asian populations, region-specific evidence is required to identify the safest and most effective protocol.

In patients with AF who have undergone percutaneous coAmuletronary intervention and require both anticoagulant and antiplatelet therapy, individualized antithrombotic management becomes even more important [93–97].

Advances in CT and echocardiographic imaging, including artificial intelligence–assisted LAA morphology analysis, have improved pre-procedural planning and device selection [98]. Integration of imaging data with clinical and hemodynamic parameters may soon enable personalized risk assessment and management in this population.

Ultimately, LAAC should be regarded not solely as an alternative to anticoagulation but as part of a comprehensive, risk-reduction strategy in AF management. Long-term follow-up data, real-world registries, and cost-effectiveness analyses will be vital for defining its evolving role in clinical practice.

## Future perspectives

The future of LAAC will be shaped by progress in imaging technology, device design, and procedural integration with other cardiovascular interventions. The growing adoption of ICE is expected to establish a fully minimally invasive workflow that avoids general anesthesia, shortens procedure duration, and reduces hospitalization [69, 70, 90]. As operator experience expands and 3D- and 4D-ICE become widely available, ICE-guided LAAC is likely to emerge as the standard approach—especially when combined with other catheter-based therapies. These advances will facilitate same-session procedures such as LAAC with AF ablation or transcatheter edge-to-edge mitral repair, improving overall procedural efficiency [37, 94].

The indications for LAAC are also expected to broaden. Although current Japanese guidelines limit its use to patients unable to tolerate long-term OAC, emerging evidence suggests potential benefit in patients with stroke despite therapeutic OAC, those with very high thromboembolic risk, and younger or dialysis-dependent populations seeking nonpharmacologic protection. As evidence accumulates, Japan is anticipated to align more closely with Western trends toward individualized, patient-centered selection criteria that balance ischemic and bleeding risks.

Ultimately, LAAC should evolve from a standalone mechanical intervention to an integral component of multidisciplinary AF management—synchronizing rhythm control, stroke prevention, and heart failure treatment within a unified continuum of care. Future directions include artificial intelligence–assisted procedural planning, polymer-coated or bioresorbable devices that enhance endothelialization, and streamlined hybrid workflows integrating PFA with LAAC. Through these innovations, LAAC is poised to become a cornerstone of comprehensive AF therapy in the coming decade.

## Conclusion

LAAC has matured into a validated stroke-prevention therapy for patients with AF who cannot tolerate long-term anticoagulation. Recent advancements—including refinement of WATCHMAN and Amulet devices, the transition from TEE to ICE guidance, and the introduction of PFA–based one-stop procedures—have enhanced procedural safety, precision, and efficiency.

Nevertheless, unresolved issues remain, including prevention of DRT, optimization of post-procedural antithrombotic therapy, and long-term evaluation of cost-effectiveness. Continued innovation in imaging, device engineering, and individualized risk stratification is expected to position LAAC as a central element of comprehensive AF management, integrated with ablation and heart failure therapy.

## Data Availability

The data generated in this study will not be shared.
